# Different transcriptional profiles of human embryonic stem cells grown in a feeder-free culture system and on human foreskin fibroblast feeder layers

**DOI:** 10.18632/aging.204282

**Published:** 2022-09-13

**Authors:** Lu Xiao, Juan Zhu, Zheng Liu, Bangyong Wu, Xiaohua Zhou, Yanxing Wei, Fei Sun, Zhijian Wang, Song Quan, Qi Li, Jun Wang, Liping Huang, Yanlin Ma

**Affiliations:** 1 Department of Obstetrics and Gynecology, Nanfang Hospital, Southern Medical University, Guangzhou 510515, Guangdong, China; 2 Hainan Provincial Key Laboratory for Human Reproductive Medicine and Genetic Research, Reproductive Medical Center, Hainan Provincial Clinical Research Center for Thalassemia, The First Affiliated Hospital of Hainan Medical University, Haikou 570102, Hainan, China; 3 Key Laboratory of Tropical Translational Medicine of Ministry of Education, Hainan Medical University, Haikou 570102, Hainan, China; 4 College of Medical Laboratory Science, Guilin Medical University, Guilin 541004, Guangxi, China; 5 Center for Molecular Development and Disease, Institute of Biosciences and Technology, Texas A&M Health Science Center, Houston, TX 77030, USA

**Keywords:** human foreskin fibroblast cells, feeder-free culture system, human embryonic stem cell, transcriptional profiles, microarray

## Abstract

Feeder cells provide an optimal microenvironment for the propagation of human embryonic stem cells (hESCs) by supplying currently known or unknown factors. However, the hESCs grown on feeder cells are not suitable for the purpose of clinical application because of the risk of contamination. In recent years, the feeder-free culture method has been developed to eliminate contamination, but some studies show that hESCs exhibit poor growth patterns in a feeder-free culture system. Regarding this phenomenon, we speculate that some genes related to hESC propagation were differently expressed in hESCs grown on feeder cells. To test this hypothesis, 3 hESC lines (NF4, NF5 and P096) were efficiently expanded in a feeder-free culture system or on human foreskin fibroblast (HFF) cells. The different gene expression patterns of hESCs in these 2 conditions were analyzed through microarrays. The results revealed that the hESCs cultured in both conditions maintained the expression of stemness markers and the ability to spontaneously differentiate into the 3 germ layers. The analysis of gene expression profiles revealed that 23 lncRNA and 15 genes were significantly differentially expressed in these two culture conditions. Furthermore, GO analyses showed that these genes were involved in such biological processes as growth factor stimuli, cell growth, and stem cell maintenance. To summarize, our study demonstrated that the hESCs grown on the HFF showed different gene expression patterns compared to those grown in a feeder-free culture system, suggesting that these differently expressed lncRNAs and genes played important roles in maintaining hESC propagation.

## INTRODUCTION

Human embryonic stem cells (hESCs) are useful tools for studying human diseases and the genetic functions of healthy organisms [1, 2]. In most current methods, hESCs are cultured on mouse or human feeder cells, which appears to be the most reliable way to maintain hESC propagation [3]. However, feeder cell-dependent cultures are often problematic because animal-derived feeder cell layers pose the risk of zoonotic disease transmission and human-tissue-based feeder layers show a risk of contamination [4]. To overcome these problems, many feeder-free culture systems for supporting hESC propagation, such as mTeSR1 (Stem Cell Technologies, Vancouver, Canada), StemPro hESC SFM (Thermo Fisher, China) and HEScGRO (Millipore, China), have recently been developed [5].

A slightly different composition supporting hESC propagation is used in each feeder-free culture system mentioned above [6]. However, the cells cultured in these systems lack cell attachment, causing cell deaths or leading to overt cell differentiation [7, 8]. In recent years, experimental evidence has indicated that feeder cell layers support the undifferentiated growth of hESCs by releasing growth factors to culture media and promoting hESCs to express a variety of unidentified factors, so as to maintain hESC attachment, differentiation and proliferation [9]. Human-tissue-derived human foreskin fibroblasts (HFFs) are the most widely used feeder cells for hESC propagation because their derivation is more practical and less invasive than other human-tissue-based feeder cells [10]. Moreover, HFFs can be cultured for up to 60 passages before senescence [11]. In the present study, we hypothesized that the transcriptional profiles of hESCs were different in feeder-dependent and feeder-free culture systems. To test this hypothesis, we evaluated the changes of gene expression in three hESC lines, namely NF4, NF5 and P096 cultured in a feeder-free culture system or on HFF feeder layers.

## RESULTS

### The culture systems maintain the pluripotency of hESCs

**Figure 1:**
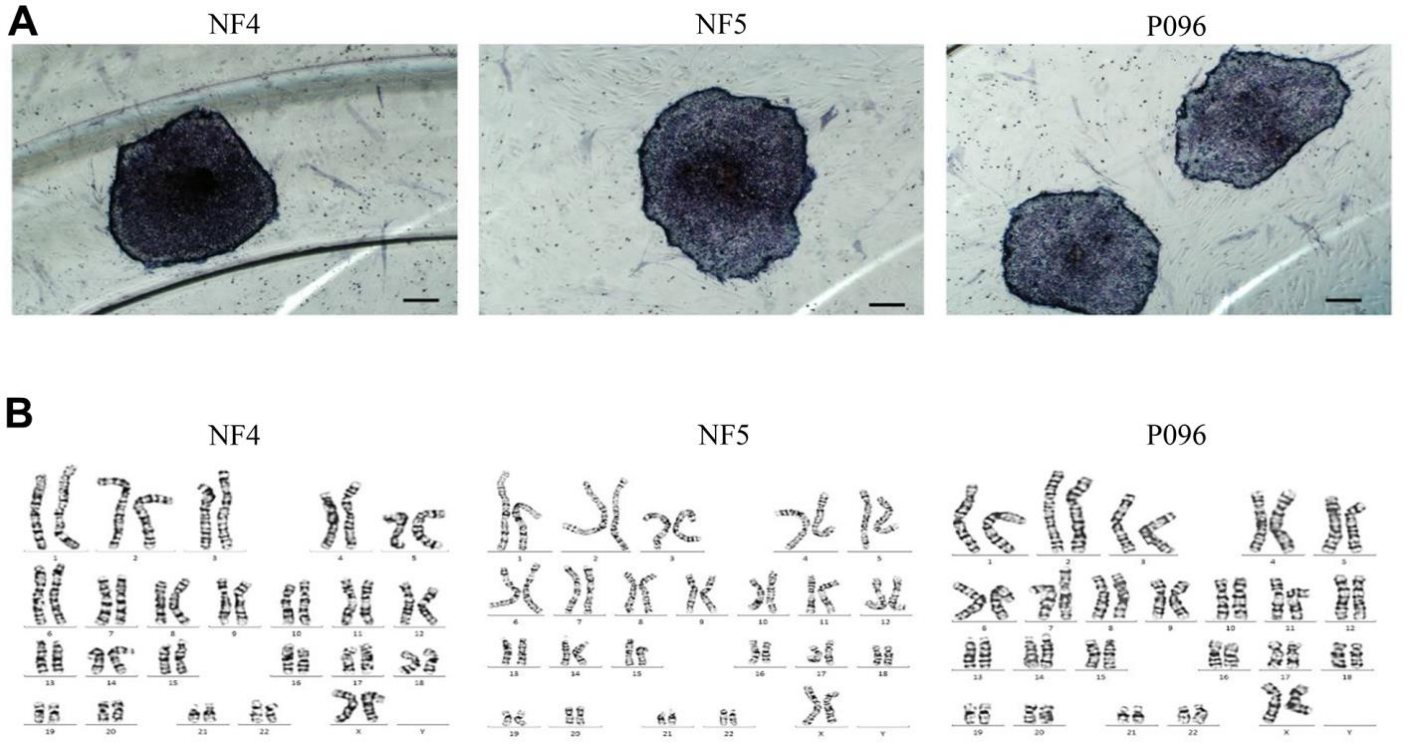
**hESCs (NF4, NF5 and P096) grown on human foreskin fibroblast.** (**A**) hESCs are positive for alkaline phosphatase staining. The scale bar was 200 μm. (**B**) Karyotype analysis revealed that these hESCs had a normal karyotype.

**Figure 2:**
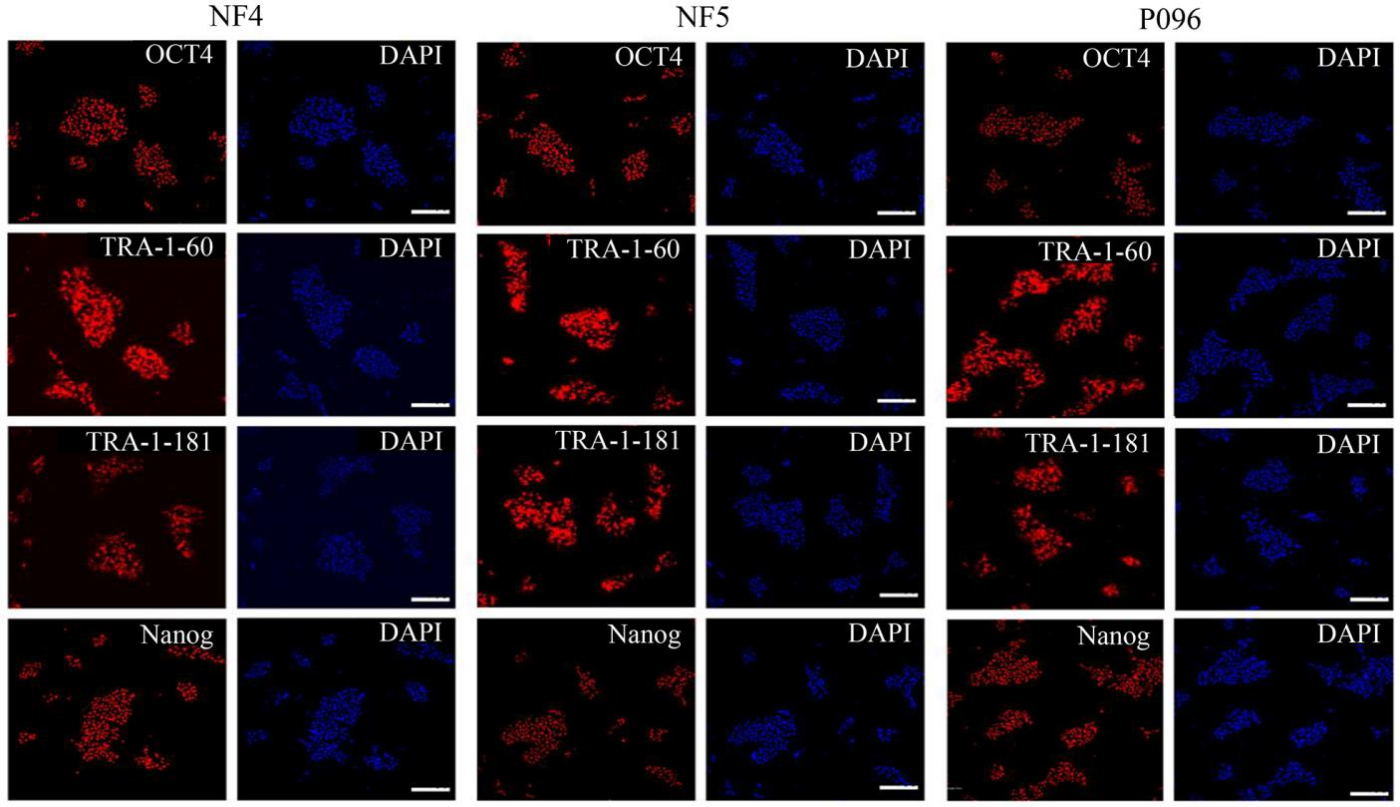
**Immunofluorescence staining.** The expressions of OCT4, Nanog, TRA-1-60 and TRA-1-181 in hESCs were detected through immunofluorescence staining. The scale bar was 100 μm.

**Figure 3:**
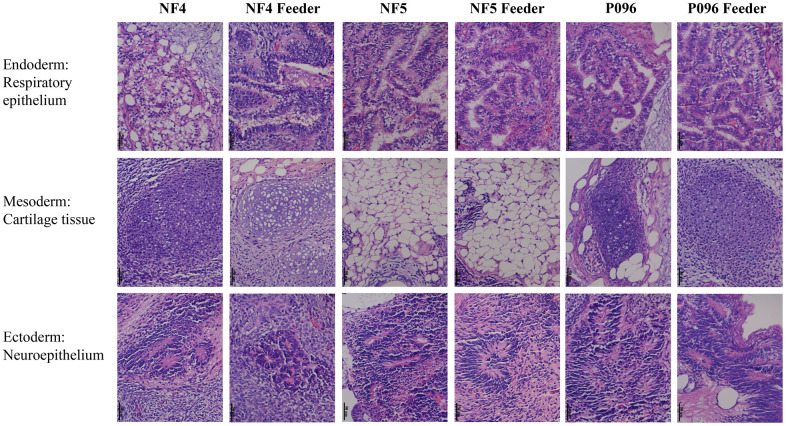
**Teratomas containing derivatives of all the 3 germ layers.** Histological evidence of germ layer differentiation in NF4, NF5 and P096 in the feeder-free culture system or on HFF feeder layers. A typical teratoma containing endoderm (respiratory epithelium), mesoderm (cartilage tissue) and ectoderm (neuroepithelium). Magnification was 400× in total.

Alkaline phosphatase staining (Figure 1A), karyotype analysis (Figure 1B) and immunofluorescence staining with pluripotent markers (OCT4, Nanog, TRA-1-60 and TRA-1-181) (Figure 2) were used to characterize 3 hESC cell lines (NF4, NF5 and P096) derived from human pre-implanted embryos. The above hESC lines were positive in both AP and immunofluorescence staining, indicating that they had the characteristics of hESCs. The results of karyotype analysis showed that these hESCs exhibited normal karyotype patterns. To illustrate the differentiation potential of NF4, NF5 and P096 in feeder-free culture systems or on HFF feeder layers, teratoma formation was performed *in vivo*, meanwhile H & E staining of teratoma sections indicated that the NF4, NF5 and P096 cells could generate teratoma containing trilineage tissues (Figure 3).

### The profiles of mRNA and lncRNA expression

**Figure 4:**
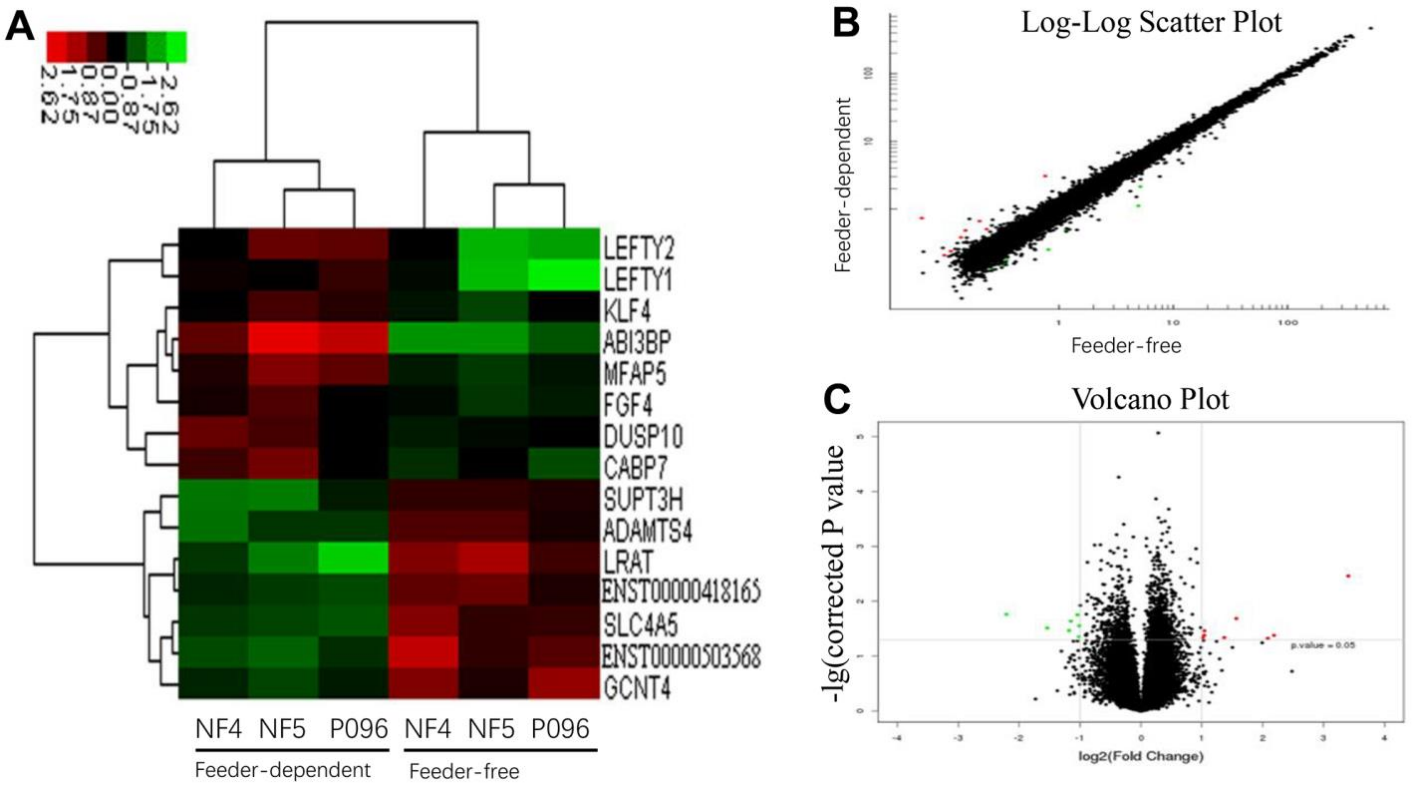
**The profiles of the differentially-expressed genes in hESCs.** (**A**) Hierarchical clustered heat maps show the Log2-transformed expression values of differentially-expressed genes in the feeder-free culture system and on human foreskin fibroblast feeder layers. Scatter plots (**B**) and volcano plots (**C**) of genes are of significantly different expressions.

**Figure 5:**
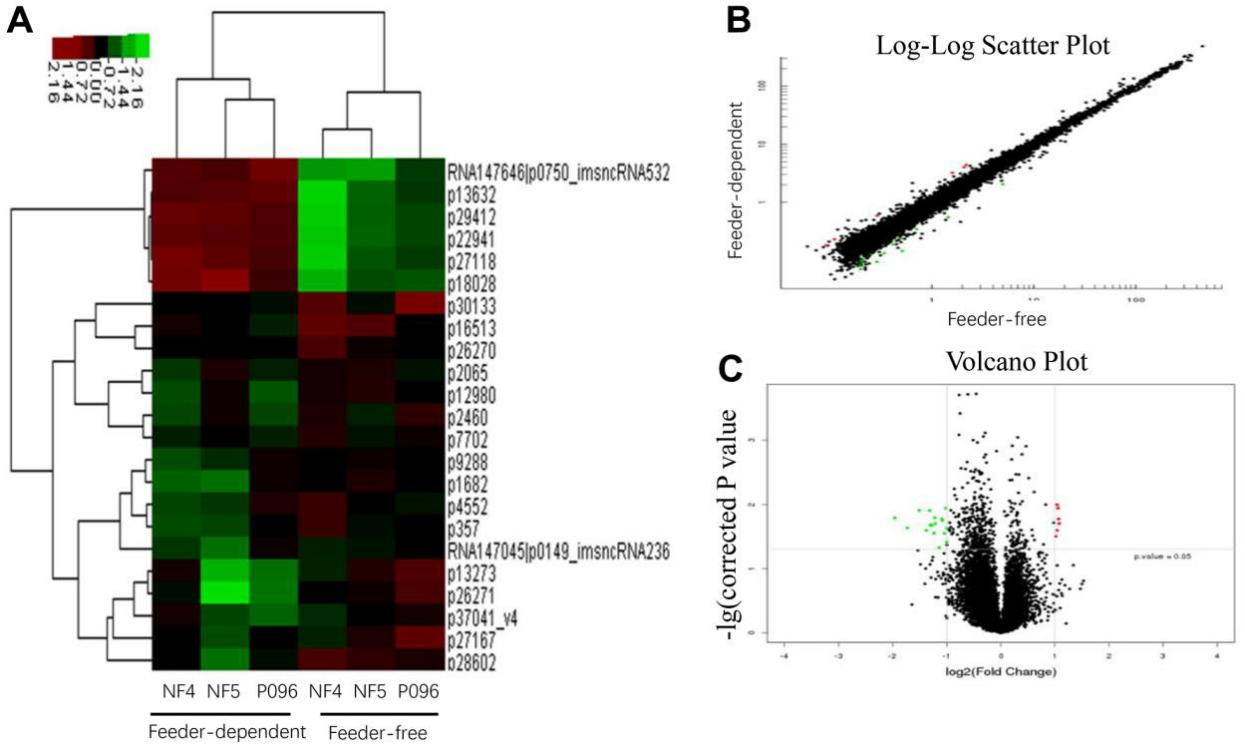
**The profiles of the differentially-expressed lncRNA in hESCs.** (**A**) Hierarchical clustered heat maps show the Log2-transformed expression values of differentially-expressed lncRNA in the feeder-free culture system and on human foreskin fibroblast feeder layers. Scatter plots (**B**) and volcano plots (**C**) of lncRNA are of significantly different expressions.

Next, we performed a gene profiling analysis as well as mRNA and lncRNA microarrays for these three hESC lines cultured in a feeder-free culture system or on HFF feeder layers. The changes in the expression were defined significant when the fold change was ≥ 2.0 with a *P* value ≤0.05. The results are summarized in Table 1. The mRNA and lncRNA expression data was then clustered using Cluster 3.0. Using dendrogram-based methods, the samples were further clustered into two subgroups through hierarchical clustering based on similar expression patterns. The results indicated that the expression of these genes and lncRNA was significantly different in 2 different culture systems. A total of 8 genes (*ABI3BP*, *LEFTY2*, *LEFTY1*, *KLF4*, *FGF4*, *DUSP10*, *MFAP5* and *CABP7*) were upregulated and 7 others (*SUPT3H*, *LRAT*, *SLC4A5*, *ADAMTS4*, *GCNT4*, *ENST00000418165* and *ENST00000503568*) were downregulated in the feeder-free culture system compared to those on HFF feeder layers (Figure 4). Also, compared with HFF feeder layers, 6 lncRNA (ENSG00000250337.1, ENSG00000250337.1, uc.332-, XLOC_004323 and XLOC_008995) were upregulated in a feeder-free culture system, while 17 others (ENSG00000224750.2, ENSG00000172965.7, ENSG00000245526.3, ENSG00000268364.1, ENSG00000259116.1, LOC100996319, ENSG00000236373.1, DQ266889, ENSG00000272033.1, ENSG00000255506.1, ENSG00000248329.1, ENSG00000268873.1, BC069804, uc.356-, ASO1967 and ENSG00000237993.1) were downregulated in a feeder-free culture system (Figure 5). Based on the genomic relationship between the lncRNAs and the protein-coding genes, lncRNA was classified as intergenic, sense, antisense, intronic and divergent lncRNA. 23 differently-expressed lncRNA was classified as 10 intergenic, 7 antisense, 3 intronic and 3 unknown lncRNAs.

**Table 1:** Summary of differentially expressed genes and lncRNAs in feeder-free culture system and on human foreskin fibroblast feeder layers.

	**Up-regulation**	**Down-regulation**	**In total**
mRNA	8	7	15
LncRNA	6	17	23

Significant differential expression was defined as probes with P≤0.05 and absolute fold change ≥2.

### Gene ontology and pathway analysis

**Figure 6:**
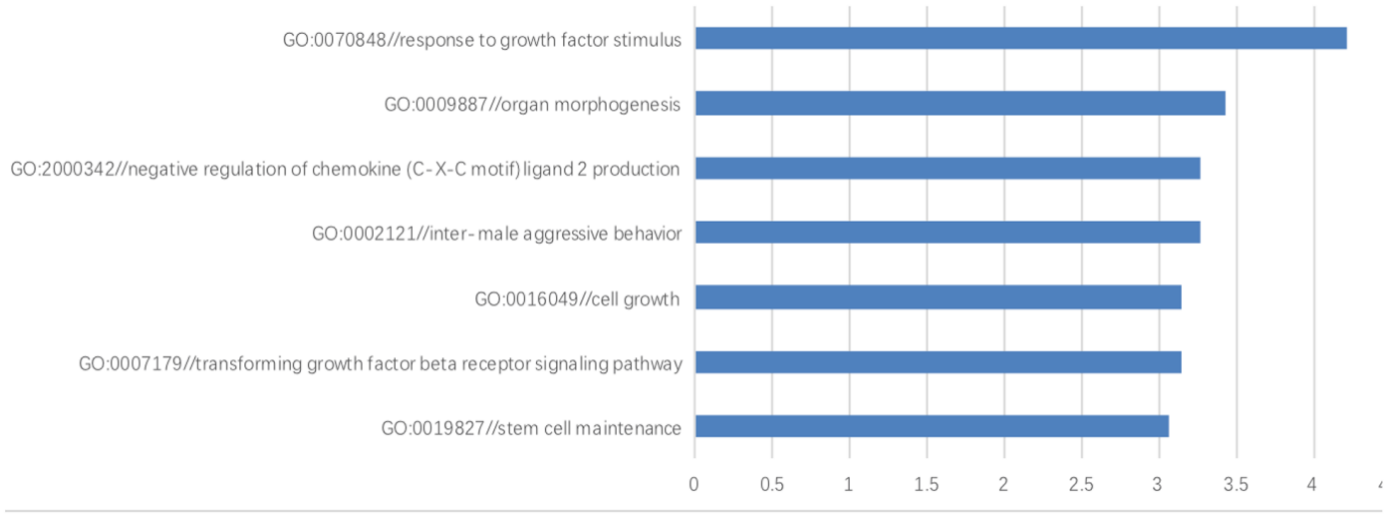
**The gene ontology enrichment analysis.** The results of gene ontology enrichment analysis revealed that these differentially-expressed genes were involved in several biological processes including growth factor stimulus, organ morphogenesis, negative regulation of chemokine (C-X-C motif) ligand 2 production, inter-male aggressive behavior, cell growth, transforming growth factor beta and stem cell maintenance.

A GO analysis was performed to investigate the biological processes, cellular components and molecular functions of those differentially-expressed genes. Compared with the hESCs grown on the HFF feeder layers, the differentially-expressed genes in the top 7 GO terms during the biological process were those involved in response to growth factor stimulus (*P*=6.165E-05), organ morphogenesis (*P*=0.0004), transforming growth factor-beta (*P*=0.0007), cell growth (*P*= 0.0007), stem cell maintenance (*P*=0.0009), inter-male aggressive behavior (*P*=0.0006) and the negative regulation of chemokine (C-X-C motif) ligand 2 production (*P*=0.0006) (Figure 6).

**Figure 7:**
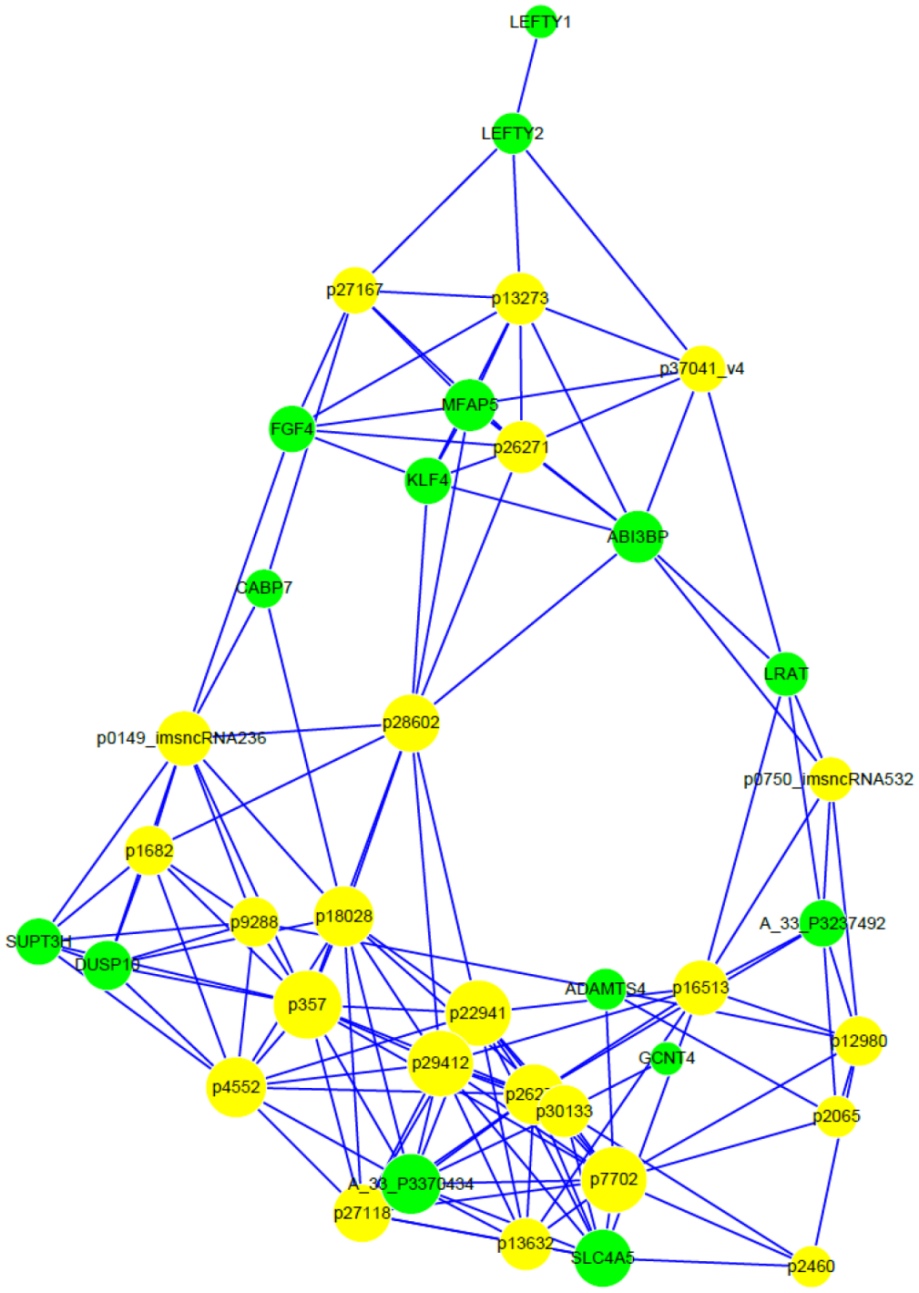
**Correlated expression networks of differentially-expressed lncRNA and genes.** Co-expression networks were constructed with differentially-expressed lncRNA and genes from 3 paired hESC lines in a feeder-free culture system vs on human foreskin fibroblast feeder layers. Pearson’s correlation > 0.99 or < -0.99, and *P* < 0.05.

Moreover, a KEGG pathway analysis performed with genes differentially expressed in differently-cultured stem cells showed the enrichment of gene categories. These target genes were significantly enriched in 3 different pathways (Table 2), of which the ‘TGF-beta signaling pathway’ was the most significant (*P*= 0.002). Next, correlated expression networks were constructed to explore the potential relationship between the differentially-expressed lncRNA and genes (Figure 7A). Uc.356-, ENST00000504246.1, uc003iol.3 and ENST00000607611.1 were identified as the node attributes of the networks, which correlated with the expression of *LEFTY2*, *ABI3BP*, *MFAP5*, *KLF4* and *FGF4* genes (Figure 7B).

**Table 2:** KEGG pathway analysis.

**Name**	**Gene symbol**	***P*-value**
ko04350 TGF-beta signaling pathway	LEFTY2; LEFTY1	0.00244515308437387
ko00512 Mucin type O-Glycan biosynthesis	GCNT4	0.0203768610313273
ko04744 Phototransduction	CABP7	0.0415987534109394
ko04745 Phototransduction - fly	CABP7	0.0487835224034567

### Validation of array-based gene expression profiles through real-time RT-PCR

**Figure 8:**
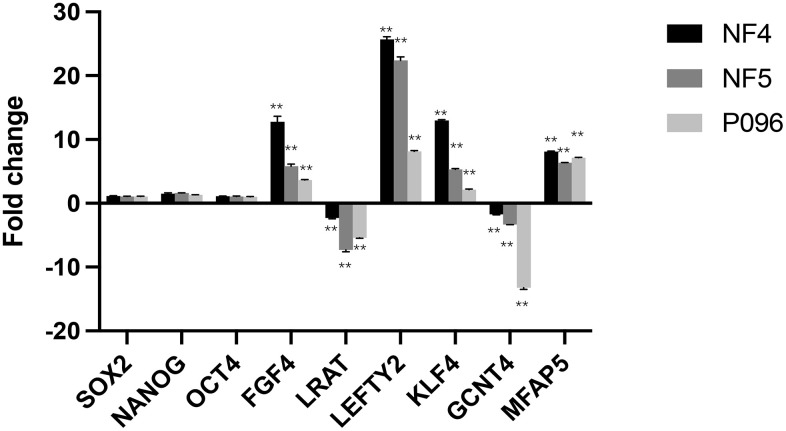
**Validation of gene-relative changes through real-time RT-PCR.** Fold changes of the gene expression in 3-line hESCs (NF4, NF5, P096) were calculated in a feeder-free culture system vs on human foreskin fibroblast feeder layers. Data was presented as mean values ± SD (n=3). The significant level was determined as *P*<0.05 using unpaired students’ test. **, *P* < 0.01.

To validate the changes mentioned above in gene expression obtained through microarray analyses, we selected 3 core pluripotent transcript factors (*SOX2*, *Nanog* and *OCT4*) and 6 differentially-expressed genes (*FGF4*, *LEFTY2*, *KLF4*, *MFAP5*, *LRAT* and *GCNT4*) for qRT-PCR validation. As is shown in Figure 8, 3 core pluripotent transcript factors showed no significant differences in the expression of the feeder-free culture system or the HFF feeder layers. The RT-qPCR confirmed the changes in the expression of those 6 genes (*FGF4*, *LEFTY2*, *KLF4*, *MFAP5*, *LRAT* and *GCNT4*) observed in the microarray analyses.

## DISCUSSION

Approaches to developing a feeder-free culture system consisting of fully defined factors will be used to significantly improve the usefulness of hESCs in human therapeutic applications [12]. In our study, a lncRNA+ mRNA human gene expression microarray analysis was used to identify differentially-expressed lncRNA and genes in a feeder-free culture system or on HFF feeder layers, so as to exploit the secreted-factors of hESCs supporting hESC propagation. The expression of 3 pluripotent transcript factors, SOX2, Nanog and OCT4, was no different, indicating that these 2 culture systems could maintain the essential characteristics of hESCs. However, we found that the expression of *ABI3BP*, *LEFTY2*, *LEFTY1*, *KLF4*, *FGF4*, *MFAP5*, *SLC4A5*, *ADAMTS4*, *DUSP10*, *CABP7*, *SUPT3H*, *LRAT* and *GCNT4* was significantly different in the feeder-free culture system and on HFF feeder layers. To predict the potential functions of these differential genes identified in this study, a GO analysis was performed using the coding genes associated with the significantly differentially-expressed mRNA. The results revealed that these genes were involved in biological processes including growth factor stimulus, transforming growth factor-beta, cell growth and stem cell maintenance, all of which were important in stem cell growth, proliferation as well as differentiation.

Amongst these genes, *LEFTY1* and *LEFTY2* controlled the balance between self-renewal and pluripotent differentiation in mouse embryonic stem cells [13]. The differentiation potential of embryonic stem cells increased due to *LEFTY1* knockdown, whereas the self-renewal of embryonic stem cells was enhanced by *LEFTY2* knockdown [14]. Interestingly, the function of *KLF4* was to activate the *LEFTY1* promoter cooperating with *Oct3/4* and *Sox2*, suggesting that a network was formed by *LEFTY1*, *LEFTY2* and *KLF4* in controlling the hESC propagation [14]. *FGF4* has been found to specifically contribute to the differentiation of hESC development at early stages [15]. The inhibition of *FGF4* signaling pathway enhanced the derivation and propagation of stem cells at undifferentiated states [16]. *ABI3BP* is an extracellular matrix protein [17], which was shown as a multifunctional autocrine/paracrine factor regulating mesenchymal stem cells in a previous report [18]. A related function of *MFAP5* is that it can remodel extracellular matrixes that may play a role in supporting hESC growth [19]. A protein that transports sodium and bicarbonate across the cell membrane as well as regulates cellular pH is encoded through *SLC4A5* [20]. *ADAMTS-4* is a metalloprotease that plays a role in aggrecan degradation in an extracellular matrix [21]. These 4 genes, namely *ABI3BP*, *MFAP5*, *SLC4A5* and *ADAMTS-4*, express proteins of extracellular matrixes, which have been widely demonstrated to support hESC propagation. The function of remaining genes in hESC growth, namely *DUSP10*, *CABP7*, *SUPT3H*, *LRAT* and *GCNT4*, are rarely reported. DUSP10 has been found to negatively regulate the inflammatory response and colorectal cancer cell growth [22]. MFAP5 is a suppressor of oral tongue squamous cell carcinoma and cervical cancer [23, 24]. The value of these factors contributes to maintaining the propagation of hESCs, which needs to be further investigated.

In recent years, accumulating documents have revealed that lncRNA was involved in the differentiation of hPSCs by affecting the level of transcription factors [25, 26]. However, the precise mechanism of how lncRNAs regulate transcription factors in hPSC propagation remains largely unknown. In the present study, 6 and 17 lncRNAs were significantly up- and down- regulated on HFF feeder layers respectively. Of the dysregulated lncRNA, we identified that 10 of them were transcribed from intergenic regions. The remaining 7 and 3 lncRNA was respectively transcribed from the antisense and intronic region. Furthermore, we employed a lncRNA-mRNA network analysis to identify interactions between differentially-expressed genes and lncRNA. Our results showed that the expression of Uc.356-, ENST00000504246.1, uc003iol.3 and ENST00000607611.1 was correlated with *LEFTY2*, *ABI3BP*, *MFAP5*, *KLF4* and *FGF4*. Interestingly, these 5 genes were identified in supporting hPSC propagation in this study, suggesting that a network controlling the hESC propagation was formed by Uc.356-, ENST00000504246.1, uc003iol.3, ENST00000607611.1, *LEFTY2*, *ABI3BP*, *MFAP5*, *KLF4* and *FGF4*.

In conclusion, we identified that 23 lncRNAs and 15 genes in hPSCs were significantly differentially expressed in the feeder-free culture system and on HFF feeder layers. In addition, our data indicated that most of these genes participated in specific biological processes contributing to hPSC propagation. Our findings here provided a piece of newfound information regarding the potential roles of these lncRNAs and genes in supporting hPSC propagation. Further research is required to elucidate the detailed molecular mechanisms underlying the action of these lncRNAs and genes.

## MATERIALS AND METHODS

### Derivation and culture of hESCs

The hESCs derived from human pre-implanted embryos and HFF cells were performed in compliance with the protocols approved by the Reproductive Study Ethics Committee of Nanfang Hospital, Southern Medical University (Research License AF/SC-08/03.2). Embryos were obtained from the donor couples undergoing *in vitro* fertilization (IVF) at the Center of Reproductive Medicine of Nanfang Hospital. Informed consent forms were signed for the participation of those couples in this study.

The blastocysts discarded in a routine IVF program were used to derive the hESC lines. The inner cell mass (ICM) was mechanically isolated with an insulin syringe needle through a stereoscope, which was then cultured on HFF feeder layers that had been mitotically inactivated with 10 μg/ml mitomycin C (M4287, Sigma, Beijing, China). HFFs were isolated from children’s foreskin after circumcision. The procedure was approved by the Reproductive Study Ethics Committee of Southern Medical University Nanfang Hospital (Research License AF/SC-08/03.2). An informed consent from the legal guardians was obtained with signed forms. Briefly, the foreskin was cut into small pieces and digested with 0.25% trypsin (Invitrogen, Beijing, China). After digestion, cells were placed into tissue culture flasks in 90% Dulbecco’s modified Eagle’s medium (DMEM; Invitrogen, China) supplied with 10% HS and 1% penicillin–streptomycin (PS; HyClone Laboratories, Inc., Logan, UT, USA). The medium was changed daily. HFFs were passaged through trypsin at a split ratio of 1:6 every 2-3 days.

The primary hESC colonies were sliced into equal-sized pieces and removed from the dish mechanically with a syringe needle through a stereoscope. The cell clusters were then transferred into new dishes containing fresh feeder cells. The hESC colonies were passed to new dishes every 4-5 days. The culture medium of hESCs was mTeSR1 (Stem Cell Technologies, Vancouver, Canada), which was replenished daily. At ten passages, 3 hESC lines (NF4, NF5 and P096) were chosen to be cultured in a feeder-dependent (HFF feeder layer) and a feeder-free culture system (matrigel-coated dish) with mTeSR1 as the culture medium (Stem Cell Technologies, Vancouver, Canada). The cells were maintained in a 37° C incubator with 5% CO_2_.

### Alkaline phosphatase staining, karyotype analysis and immunofluorescence staining

AP staining, karyotype analysis and immunofluorescence staining were performed on hESCs with a passage between 6 and 8. AP staining was carried out using an AP detection kit (Millipore, Beijing, China) according to the manufacturer’s protocol. Briefly, cells were fixed in 4% paraformaldehyde for 20 min at room temperature. After washing thrice with 1×PBS, cells were treated in the ALP buffer for 5 min and stained in BCIP (5-bromo-4-chloro-3-indolyl-phosphate) as well as NBT (nitro-blue-tetrazolium) for another 15 min. The images of AP staining were captured with a Zeiss microscope.

For karyotype analysis, cells were first treated with 0.2 μg/ml colcemid (Gibco Invitrogen, Beijing, China) for 130 min. The hESC colonies were mechanically removed from the feeder layers through a stereoscope after washing twice in DMEM/F12. The cells were incubated in 0.4% kalium chloratum for 20 min at 37° C and then fixed in methanol: acetic acid (3:1, v/v) for 3 min, followed by Giemsa staining. 20 cells per group were randomly selected for the karyotype analysis.

For immunofluorescence staining, cells were washed in 1×PBS (Gibco) twice before and after being fixed in 4% paraformaldehyde for 20 min at room temperature. Cells were then permeabilized in 0.3% triton X-100 (Sigma, Beijing, China) for 1 h, followed by a block in 10% goat serum in PBS. After then, cells were incubated with primary antibodies of interest (anti-OCT4 antibody, CST; anti-Nanog antibody, CST; anti-TRA-1-60 antibody, CST; anti-TRA-1-181 antibody, CST) at 4° C overnight, followed by another incubation with second antibodies (Goat anti-Mouse, Alexa Fluor 568, Abcam) at room temperature for 1 h. DAPI was used for nuclear staining. Immunostaining images were captured with a Leica DMI6000B microscope (Leica Microsystems GmbH, Germany).

### Teratoma formation assay

About 3×10^6^ hESCs were suspended in 25% matrigel with DMEM/F12 (1:3), and then injected subcutaneously into immunodeficient nude mice, which were humanely sacrificed 8 weeks after injection. The teratomas were excised and processed for hematoxylin-eosin staining (H & E). Tissues were fixed in 4% paraformaldehyde for 24 h, followed by dehydration in gradient ethanol (70%, 85%, 95% and 100%) and paraffin embedding. For H&E staining, 6 micrometer sections were deparaffinized twice in xylene (each for 5 min), rehydrated in gradient ethanol (100%, 90% and 70%), stained with hematoxylin for 4 min, washed in ddH_2_O for 5 min, treated with 1.5% hydrochloric acid-75% ethanol for 10 s and washed in ddH_2_O for 5 min, which was followed by PBS for 2 min, they were then stained with eosin for about 20 s and dehydrated in gradient ethanol as well as xylene, after which they were placed in xylene and neutral resin mounting medium. All animal protocols were approved by the ethics committee at Hainan Medical University.

### Sample collection and RNA extraction

The hESCs on a HFF feeder layer with a passage of 3 (totally 13 passages) were mechanically removed from feeder layers through a stereoscope after washing twice in DMEM/F12, after which they were collected concurrently in a trizol (Invitrogen, Beijing, China) and stored at -80° C. Total RNA was extracted from the frozen trizol block and purified using NucleoSpin RNA clean-up (Macherey-Nagel, Duren, Germany). The concentration and purity of RNA were determined by a NanoDrop ND-2000 spectrophotometer (NanoDrop Technologies, Thermo Scientifics, USA), and its integrity was evaluated through formaldehyde denaturing gel electrophoresis. Only the RNA samples with a total RNA quantity ≥ 1 μg and an A260/ 280 ratio ≥ 2.0 showing clear bands of 28 s as well as 18 s rRNA with a brightness ratio of near 2:1 in the formaldehyde denaturing gel were used for the microarray analysis.

### Microarray and analysis of gene expression

A total 200 ng of RNA was amplified and reversely transcribed to double-stranded cDNA and subsequently labelled through fluorescent dye (Cy3) according to the manufacturer’s protocol for CapitalBio cDNA Amplification and Labeling Kit (CapitalBio, Beijing, China). The labelled cDNA was then purified and hybridized to Agilent Human lncRNA + mRNA Array V3.0 (Agilent, USA). Images were scanned with an Agilent microarray scanner (G2565CA) and analyzed using Agilent feature extraction (v10.7). The raw data was summarized and normalized using GeneSpring (Agilent V12.0). After completing quantile normalization of the raw data, differentially expressed lncRNA and genes were identified using threshold values of ≥2- and ≤−2-fold changes and a Benjamini-Hochberg corrected *P* value of ≤0.05. Next, the Molecular Annotation System (CB-MAS) V3.0 (http://bioinfo.capitalbio.com/mas3/) was used to perform gene ontology and pathway analysis. The GO terms consisted of three families: biological process (BP), cellular component (CC) and molecular function (MF).

In addition, signaling pathway analysis of these genes was performed according to KEGG PATHWAY Database (http://www.kegg.jp/kegg/pathway.html), which provided information about the significant enrichment of genes with differential expression. Finally, the differentially-expressed lncRNA and genes with a Pearson correlation coefficient no. < 0.99 were chosen to draw a co-expression network. The target prediction was performed based on their close correlations (the minimum Pearson’s correlation coefficient was -0.7) to a group of differentially-expressed protein-coding genes.

### Real-time PCR

The total RNA of these 3 hESC lines was isolated using a RNeasy mini kit (QIAGEN, Beijing, China) and was reversely transcribed using a reverse transcription system kit (Promega, USA) in accordance with the manufacturer’s instructions. Real-time PCR was performed with the oligos for the selected genes with Brilliant III Ultra-Fast qPCR Master Mix (Agilent Technologies, USA). The real-time PCR conditions are shown below: 95° C for 2 min; 30s at 95° C and 1 min at 60° C with a total of 45 cycles; melt curve stage (95° C for 15 s, 55° C for 1 min and 90° C for30 s). GAPDH was used as an internal control, and the 2^−ΔΔCT^ method was used to calculate the mRNA fold changes (feeder-free cultured hESCs/feeder-dependent cultured hESCs). The primers used in this study are listed in Table 3.

**Table 3:** The primers used in this study.

**Genes**		**Primer**
*SOX2*	Forward	GGTTACCTCTTCCTCCCACTCC
	Reverse	CCCTCCCATTTCCCTCGTTT
*NANOG*	Forward	ACCTATGCCTGTGATTTGTGG
	Reverse	AGTGGGTTGTTTGCCTTTGG
*OCT4*	Forward	GGCCTGCATGAGGGTTTCT
	Reverse	CCCCTGAGAAAGGAGACCCA
*FGF4*	Forward	CTCGCCCTTCTTCACCGATG
	Reverse	GTAGGACTCGTAGGCGTTGTA
*LEFTY2*	Forward	TGGACCTCAGGGACTATGGAG
	Reverse	CCGAGGCGATACACTGTCG
*KLF4*	Forward	CGGACATCAACGACGTGAG
	Reverse	GACGCCTTCAGCACGAACT
*MFAP5*	Forward	TGAATGATCCCGCTACAGATGA
	Reverse	TCCTTACAGACAAGACGAGAGC
*LRAT*	Forward	TGATGCCCGACATCCTGTTG
	Reverse	ATGTTAGCTCCGTAGGCGAAG
*GCNT4*	Forward	TCCCTAAGTACCTCGCCTTTT
	Reverse	GCTCCTGTTCATAGATACCCGAA
*GAPDH*	Forward	CATGTTCGTCATGGGTGTGAACCA
	Reverse	AGTGATGGCATGGACTGTGGTCAT

### Statistical analysis

Results were presented as mean ± SD with at least 3 biological repeats using a GraphPad prism. Significance level was determined by unpaired two-tailed students’ t tests, and *P*< 0.05 was considered statistically significant.
